# Genetic Diversity and Resistome Analysis of *Campylobacter lari* Isolated from Gulls in Croatia

**DOI:** 10.3390/antibiotics12081310

**Published:** 2023-08-12

**Authors:** Luka Jurinović, Sanja Duvnjak, Andrea Humski, Biljana Ječmenica, Louie Thomas Taylor, Borka Šimpraga, Fani Krstulović, Tajana Amšel Zelenika, Gordan Kompes

**Affiliations:** 1 Laboratory for Bacteriology, Croatian Veterinary Institute, Poultry Centre, 10000 Zagreb, Croatia; jurinovic@veinst.hr (L.J.); b_simpraga@veinst.hr (B.Š.); f_krstulovic@veinst.hr (F.K.); t_amsel-zelenika@veinst.hr (T.A.Z.); 2 Laboratory for Bacterial Zoonoses and Molecular Diagnostics of Bacterial Diseases, Department for Bacteriology and Parasitology, Croatian Veterinary Institute, 10000 Zagreb, Croatia; marjanovic@veinst.hr; 3 Laboratory for Food Microbiology, Department for Veterinary Public Health, Croatian Veterinary Institute, 10000 Zagreb, Croatia; 4 Assocciation BIOM, 10000 Zagreb, Croatia; louie.taylor@biom.hr; 5 Laboratory for General Bacteriology and Mycology, Department for Bacteriology and Parasitology, Croatian Veterinary Institute, 10000 Zagreb, Croatia; kompes@veinst.hr

**Keywords:** antimicrobial resistance, wild birds, prevalence, MLST, antibiotics

## Abstract

*Campylobacter lari* is a thermotolerant bacterium that sporadically causes gastrointestinal diseases in humans and can be found in wildlife and the environment. *C. lari* is an understudied species, especially in wild birds such as gulls. Gulls are potentially good carriers of pathogens due to their opportunistic behavior and tendency to gather in large flocks. During winter and their breeding period, 1753 gulls were captured, and cloacal swabs were taken to be tested for the presence of *C. lari*. From isolated bacteria, the DNA was sequenced, and sequence types (ST) were determined. Sixty-four swabs were positive for *C. lari*, and from those, forty-three different STs were determined, of which thirty-one were newly described. The whole genome was sequenced for 43 random isolates, and the same isolates were tested for antimicrobial susceptibility using the broth microdilution method to compare them to WGS-derived antimicrobial-resistant isolates. All the tested strains were susceptible to erythromycin, gentamicin, and chloramphenicol, and all were resistant to ciprofloxacin. Resistance to ciprofloxacin was attributed to a *gyr*A_2 T86V mutation. Genes connected to possible beta-lactam resistance (*bla*OXA genes) were also detected.

## 1. Introduction

Campylobacteriosis is the leading gastrointestinal infection in the European Union [1]. Thermophilic *Campylobacter* species are responsible for the majority of human cases, and although *C. jejuni* and *C. coli* are the most represented species in human epidemiology, some human infections are caused by *C. lari* [1]. In humans, *C. lari* is most often associated with enteric infections [2], but it can cause bacteremia, especially in immunocompromised persons [3,4,5], urinary tract infections [6], purulent pleurisy [7], arthritis, and osteomyelitis [8]. While it was first isolated from feces of a symptomless 6-year-old boy, most *C. lari* isolates are from gulls, particularly Herring Gulls, *Larus argentatus*, and Black-headed Gulls (BHG), *L. ridibundus,* [9]. After the species was described, *C. lari* has been found in different hosts, such as crows [10], shellfish [11], other wild birds [12,13,14], and farm animals [15,16,17], and also in environmental samples [18,19,20].

Only 462 isolates of *C. lari* have been genotyped using MultiLocus Sequence Typing (MLST) and are available on the PubMLST database (https://pubmlst.org, accessed on 27 July 2023) [21]. Most of them (83%) were isolated from different species of wild birds (159) and shellfish (143) and humans (78). The rest of the isolates (17%) come from a variety of sources in the environment (fresh and marine water, soil, etc.).

Gulls are one of the most common birds in human surroundings. Although they are “known” as seabirds, many gull species are opportunistic and form huge flocks, while feeding on rubbish tips, even far inland. More than 10,000 birds from all over Europe can be seen in the winter feeding on a Zagreb rubbish tip [22,23]. Also, roosting and sleeping places are usually used by a large number of birds, and therefore, are a great opportunity for the circulation of different pathogens.

The aim of this study was to determine the prevalence of *C. lari* found in different gull species that occurin Croatia, its molecular characterization, and the antimicrobial susceptibility of isolates. Additionally, in this study, we aim to compare the phenotypic and genotypic resistances of *C. lari* strains. In Croatia, *C. lari* has only been studied in shellfish [24], and in general, it is still under-researched especially in wildlife; therefore, this is the first study conducted on gulls.

## 2. Results

A total of 1753 gulls from five species were captured in Croatia over a five-year period (2017–2022) in order to study the occurrence of *C. lari.*

During the winter months (November–March), gulls from various species were captured in three rubbish tips in Croatia: Yellow-legged Gulls (YLG), *L. michahellis* (*n* = 484); BHG (*n* = 607); Caspian Gulls, *L. cachinanns* (*n* = 17); Herring Gulls (*n* = 2) and Common Gulls, *L. canus* (*n* = 33). During the breeding season, 610 adult and juvenile gulls were captured in 15 breeding colonies (14 YLG colonies (*n* = 600) and 1 BHG breeding colony (*n* = 10)). 

There were sixty-four positive swabs for *C. lari,* mostly from YLG (54) and BHG (7), while two positive swabs were from Caspian Gulls, and one was from a Common Gull. The overall prevalence was 3.65%, with the highest isolation rate found for the Caspian Gulls (11.76%), followed by the YLG (4.98%) and Common Gulls (3.03%), while the lowest rate was for the BHG (1.13%).

MLST was performed on all the isolates, and all but four gave full sequence-type (ST) profiles. In this study, 43 different STs were identified, 31 of which were newly described (159, 160, 161, 222, 223, 224, 225, 226, 227, 228, 229, 262, 295, 296, 297, 298, 299, 306, 312, 313, 314, 315, 316, 317, 318, 319, 320, 322, 325, 326, and 327). Furthermore, 15 new alleles were described: *adk* 128; *atpA* 121, and 152; *glnA* 104, 120, and 121; *pgi* 184 and 190, *pgm* 147 and 172; *tkt* 123, 124, 127, 159, and 165. Most of the STs were detected only once (Table 1).

The distributions of MIC values among the *C. lari* isolates from the gulls’ cloacal swabs are shown in Table 2, while MIC_50_/MIC_90_ values are shown in Table 3.

All the *C. lari* strains isolated from the gulls were susceptible to erythromycin, gentamicin, and chloramphenicol (Table 2). Regarding ertapenem and tetracycline, only one isolate was resistant (2.3%). All the *C. lari* isolates in our study were found to be resistant to ciprofloxacin (Table 3). The highest MIC_50_ and MIC_90_ values were observed for ciprofloxacin (8 mg/L). The MIC_50_ and MIC_90_ values for chloramphenicol, erythromycin, tetracycline, and ertapenem were set to the lowest tested antimicrobial concentration, while the MIC_50_ and MIC_90_ value for gentamicin was 1 mL/L (Table 3).

### 2.1. Genotypic Determination of Antimicrobial Resistance (AMR)

The testing of ciprofloxacin, as one out of the six antimicrobial agents included in this study, showed genotypic and phenotypic resistance. A resistance to ciprofloxacin was detected in all the *C. lari* isolates carrying a *gyrA*_2 T86V mutation. The genes connected to possible beta-lactam resistance (*bla*OXA genes) were often identified in the tested isolates; however, as far as we know, none of those are directly connected to a specific antimicrobial beta-lactam agent. Two different *bla*OXA genes were identified; *bla*OXA-493 gene was identified in thirty-three samples (76.7%), and *bla*OXA-518 was identified in three samples (7.0%).

### 2.2. Comparison between Phenotypic and Genotypic AMRs

The phenotypic and genotypic antimicrobial predictions were correlated in the case of ciprofloxacin, where all the tested strains showed phenotypic and genotypic AMRs. One strain showed a phenotypic resistance to tetracycline and ertapenem, but not a genotypic one. In total, one strain out of the forty-three tested (2.3%) on six different antimicrobial agents showed two discordances between the phenotypic and genotypic results.

## 3. Discussion

Most of the samples included in the present study belonged to two species of gulls (YLG and BHG), and there was a great discrepancy in the isolation rates of *C. lari* between these two species (4.98% in YLG and 1.13% in BHG). One of the main ecological differences between these two species is their breeding habitat. While YLG mostly breed in marine habitats (mostly on rocky islets and sea cliffs), BHG mostly use freshwater habitats (mostly islands in lakes or wetlands) [26]. It is known that *C. lari* can tolerate a much higher concentration of NaCl than other thermotolerant species of the same genus can [27,28]. This is in accordance with published data on the prevalence of *C. lari* in different gull species; it appears higher to be in marine species, such as 3% in Herring Gulls [29] and 3.30% in YLG [30], and it is lower in freshwater species, such as 1.02% in BHG [31].

**Figure 1:**
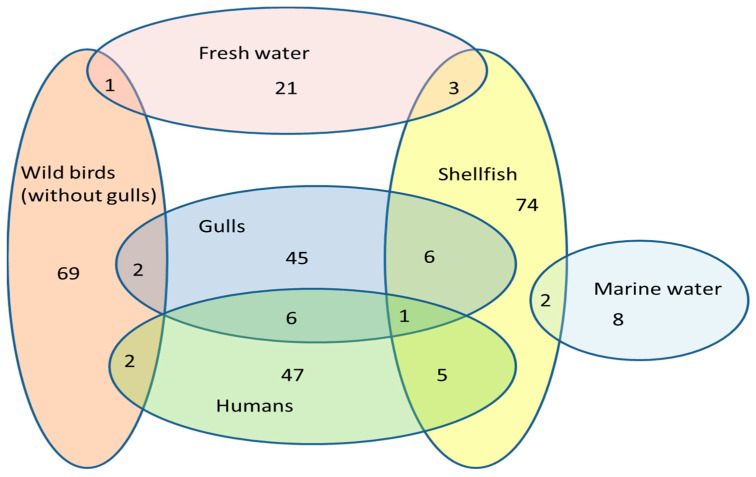
Venn diagram of *C. lari* STs from PubMLST database, including STs described in this study. Most of the STs are fitted in six groups. Nineteen isolates (belonging to nineteen different ST’s) were not taken in this analysis as the source was either not specified (e.g., “soil”, “environmental water”) or was too scarce to affect analysis (e.g., dog (*n* = 3); horse (*n* = 1); bat (*n* = 1)).

The facts that only 462 isolates of *C. lari* (as opposed to more than 80,000 *C. jejuni* or more than 20,000 *C. coli* isolates) were listed in the PubMLST database and 330 different STs were described (including isolates from this study) highlight how understudied this organism is [21]. Also, having 31 newly described ST (compared to the 158 STs described before this study) out of 64 isolates supports this statement, but it also shows the great diversity of the isolates [21]. Only 20 isolates from this study belong to the previously described STs. Four of the isolates from this study share the same STs with only one isolate from humans, four share STs with the gull isolates, two share STs with the human and shellfish isolates, one is shared with shellfish, and one is shared with Brent Geese (*Branta bernicla*) [21]. Unfortunately, the species names of wild birds are not always provided in databases, and it is not possible to link them to specific (i.e., marine) environments. If we exclude them from being a part of the marine environment analysis of the PubMLST collection of *C. lari* STs (Figure 1), this supports the fact that *C. lari* is a more halophilic bacterium than other *Campylobacter* species can, as most of the described STs (64.1%) are from shellfish, gulls, and marine water.

The most prevalent resistant gene determined in this study was the quinolone resistance *gyrA*_2 T86V gene (100%). This Thr-Val 86 substitution in the GyrA protein has been previously reported only in the *C. lari* group [32,33,34], and as it cannot be acquired due to a single point mutation, it is considered that this is the sequence of an intrinsically resistant organism [32]. Two known β-lactam resistance genes (*bla*OXA variants) were also detected: *bla*OXA-493 gene was identified in thirty-three samples (76.7%), and *bla*OXA-518 was identified in three samples (7.0%). The presence of the *bla*OXA-493 gene as the predominant β-lactamase coding gene in *C. lari* has previously been described [34,35]. Both *bla*OXA-493 and *gyrA*_2T86A were detected in a total of 33 isolates (76.7%). The authors of earlier studies found that the combination of these two genes is exclusively found in the *C. lari* group [34]. Regarding the *bla*OXA-518 gene, it has been described earlier for *C. coli* [35] and in *C. lari* (GenBank: Accession No. NG_049792, National Center for Biotechnology Information (NCBI); online: https://www.ncbi.nlm.nih.gov/nuccore/NG_049792.1, accessed on 20 July 2023, unpublished), and it belongs to the OXA-493-like subfamily, with 99.6% similarity. It differs from OXA-493 due to a Gly-Asp 72 substitution. However, functional information about the OXA-493-like subfamily is not available [36].

The overall correlation rate between the WGS-based genotypic prediction and phenotypic resistance is 95.5%.

The discordances in the results included one strain showing a phenotypic resistance to tetracycline and ertapenem. There are no known or thus-far documented mutations that were found in the investigated genome. 

Until now, a genotypic resistance to fluoroquinolones in *Campylobacter* species has been explained by the presence of different *gyrA* gene mutations. Thus far, mutations that correlate with a genotypic resistance to fluoroquinolones also present different levels of resistance, making it possible for strains with a certain mutation to be phenotypically susceptible, and vice versa [37]. Moreover, detected resistant determinants do not always confer a resistant phenotype. These findings can be explained by the existence of multiple mechanisms of resistance to antibiotics, which include a decrease in outer membrane permeability and efflux systems, and detected modifications and mutations [38]. 

Li et al. (2017) pointed out, after conducting the integrated genomic and proteomic analysis of chloramphenicol resistance in *C. jejuni,* that this integrated approach is the key to understanding AMR in its fullness. Often, there are many mechanisms in action, not just mutations of certain genes [39]. In this study, again, WGS proved to be a good starting tool for comprehensive AMR characterization that is highly concordant with phenotypic AST, and also, a great tool for quick outbreak investigations. This is especially true in cases like these, where information about AMR in *C. lari* is extremely lacking.

At the beginning of this study, it was emphasized that *C. lari* is widespread across multiple hosts and environmental samples. The authors of previous research have also pointed out that the role of *C. lari* and other *Campylobacter* species, such as *C. concisus*, *C. ureolyticus,* and *C. upsaliensis*, in human and animal diseases is underappreciated. Because of this, these species are known as ‘’emerging *Campylobacter* species’’ [40].

The fact that *C. lari* is isolated from such a wide range of habitats and its resistance to the fluoroquinolones class of antimicrobials may classify it as a reservoir of resistant gene material. Also, this supports the importance of this emerging underestimated species as a potential pathogen.

## 4. Materials and Methods

### 4.1. Sampling and Campylobacter Isolation

In the period from 22nd January 2017 to 31st May 2022, gulls were captured in Croatia in order to test their cloacal swabs for the presence of *C. lari*. During the breeding season, the gulls were caught in bird colonies using “walk in” traps set up on nests, and during the non-breeding season, they were caught with cannon net on rubbish tips. Cloacal swabs were taken and stored in Amies transport medium with charcoal (Merck, Darmstadt, Germany) to prolong the vitality of the microorganisms. After collection, the samples were transported in cool boxes at 4–8 °C and examined within a maximum of 48 h of sampling. The detection of *Campylobacter* spp. was conducted according to the standard EN ISO 10272-1 method [41], and species determination was performed using multiplex PCR [42].

Briefly, each swab was added to the liquid enrichment medium (Bolton broth; Oxoid, UK). After incubation in a microaerobic atmosphere (CampyGen; Oxoid, UK) at 37 °C for 4–6 h, and then at 41.5 °C for 44 h, a loopful (10 µL) of the enrichment cultures were streaked on the surface of each of the two selective plating mediums, modified Charcoal Cefoperozone Deoxycholate agar (mCCD agar; Oxoid, UK) and CampyFood ID agar (CFA; BioMerieux, France). Selective solid media were incubated at 41.5 °C in a microaerobic atmosphere and examined after 44 h to detect the presence of suspect *Campylobacter* colonies. All of the presumptive *Campylobacter* colonies were examined for morphology and motilityand sub-cultured on a Columbia agar (Oxoid, UK) with 5% sheep blood (Biognost, HR) for further confirmation (the microscopic verification of the characteristic morphology and motility, the detection of oxidase activity, and an aerobic growth test at 25 °C). 

### 4.2. MLST

Using the available research funding, whole genomes were sequenced from 43 randomly selected isolates. Extracted DNA was prepared according to the instructions of MicrobesNG (Birmingham, UK) and sent there for sequencing. The sequencing was conducted using an Illumina platform with a 250 bp paired-end output. The results were obtained as raw trimmed reads and assembled as fasta files. Sequence types (ST) were determined using the WGS-MLST plug-in in BioNumerics 8.1.1 version (BioMerieux, Applied Maths, Sint-Martens-Latem, Belgium).

The other 21 isolates were sequenced according to Miller et al. [43]. PCR products were sequenced at Macrogen Europe (The Netherlands). Sequences were edited using BioEdit software (version 7.2.5.). STs were determined using the *Campylobacter* multilocus sequence typing website (https://pubmlst.org/campylobacter/, accessed on 27 July 2023) sited at the University of Oxford [21].

### 4.3. Antimicrobial Susceptibility Testing

Antimicrobial susceptibility testing (AST) was conducted on 43 isolates that had their whole genome sequenced using the broth microdilution method according to the European Committee on Antimicrobial Susceptibility Testing (EUCAST) [44] recommendations for fastidious microorganisms. Briefly, the isolates were recovered from freezer stocks (Tryptic soy broth with 20% glycerol, −80 °C) and incubated overnight on blood agar supplemented with 5% sheep blood in a microaerobic atmosphere. AST was carried out on EUCAMP3 microplates (Sensititer, Trek Diagnostic Systems Ltd. East Grinstead, West Sussex, RH19 1XZ, UK). Mueller–Hinton broth was used for the preparation of 0.5 McFarland solution, and the inoculum was made by adding 100 µL of initial solution in 11 mL of cation-adjusted Mueller–Hinton broth supplemented with 5% lysed horse blood and 20 µg/L β-NAD. Microplates were incubated in a microaerobic environment at 41 °C for 24 h. The susceptibility to chloramphenicol (CHL; 2–64 mg/L), erythromycin (ERY; 1–512 mg/L), ciprofloxacin (CIP; 0.12–32 mg/L), and tetracycline (TET; 0.5–64 mg/L) was determined using EUCAST *C. coli* epidemiological cut-off values (ECOFFs), while for ertapenem (ERTA 0.12–4 mg/L) and gentamicin (GEN; 0.25–16 mg/L), the European Food Safety Authority (EFSA) cut-off values were used, as there are no available data from the EUCAST [25]. Reference strains *C. jejuni* ATCC 33560 and *Staphylococcus aureus* ATCC 29213 were used for quality control.

### 4.4. Genomics

#### 4.4.1. Whole-Genome Sequencing Data Analysis

Basic bioinformatics analysis was conducted by MicrobesNG (Birmingham, UK). All the obtained reads were put through a standard analysis pipeline. The closest available reference genome was identified using Kraken, and the reads were mapped to this using BWA-MEM (Burrows-Wheeler Aligner) to assess the quality of the data. A de novo assembly of the reads was obtained using SPAdes, and the reads were mapped onto the resultant contigs, again using BWA mem to obtain more quality metrics. Also, automated annotation was performed using Prokka.

All but five samples were de novo assembled with a genome size of approximately 1.5 Mb and a GC content of around 30%. Samples 1c5, 71, 175, 176, and 266 were inter-species-contaminated, but we used reference mapping (reference sequence: CP0000932) to isolate only the *C. lari* sequences.

#### 4.4.2. Antimicrobial Resistance

The WGS-derived antimicrobial resistance (AMR) was analyzed for de novo assemblies using the publicly available service ResFinder 4.1 at https://cge.food.dtu.dk/services/ResFinder (accessed on 10 July 2023) provided and curated by the Center for Genomic Epidemiology. We analyzed 43 assembled *C. lari* genomes for chromosomal point mutations (also for all unknown mutations) with a 98% threshold for %ID and 80% minimum length and acquired antimicrobial resistance genes using the same restrictions.

#### 4.4.3. Genotypic–Phenotypic Comparisons 

The WGS-derived AMRs were compared to the results of in vitro AST for six clinically relevant antimicrobial agents (erythromycin, ciprofloxacin, tetracycline, gentamicin, chloramphenicol, and ertapenem).

The concordance between the methods was determined by comparing the genotypic detection of known resistance determinants against the phenotypic susceptibility results of each strain at a concentration equal to the ECOFF described by the EUCAST and EFSA [25,44].

Major errors were classified as those instances where a strain was predicted to be resistant to the detection of an AMR determinant in the genome, but was phenotypically susceptible. Very large errors were classified as those instances where a strain was predicted to be susceptible by the absence of an AMR determinant in the genome, but was phenotypically resistant.

## Figures and Tables

**Table 1:** List of sequence types (ST) (*n* = 43) of *Campylobacter lari* found in five different gull species sampled in Croatia (*n* = 1753). New STs and alleles are printed bold.

ST	*adk*	*atpA*	*glnA*	*glyA*	*pgi*	*pgm*	*tkt*	Number of Isolates	Source Species
8	7	1	1	1	1	3	2	3	*Larus michahellis* (2) *Larus ridibundus* (1)
68	1	6	1	16	1	1	1	4	*Larus michahellis* (3) *Larus ridibundus* (1)
69	2	1	1	2	76	3	33	1	*Larus michahellis*
73	6	5	1	1	69	1	6	1	*Larus michahellis*
77	7	1	1	53	1	3	2	3	*Larus michahellis*
127	84	6	1	1	4	1	36	1	*Larus michahellis*
137	90	5	1	77	3	5	6	2	*Larus michahellis*
158	103	65	1	1	1	3	44	1	*Larus michahellis*
**159**	7	57	1	1	4	1	6	1	*Larus cachinnans*
**160**	6	2	1	1	1	1	6	1	*Larus canus*
**161**	103	2	1	1	1	3	44	2	*Larus michahellis*
165	4	57	1	2	1	1	2	1	*Larus michahellis*
168	8	6	1	1	1	1	2	1	*Larus cachinnans*
**222**	8	6	1	1	1	1	6	1	*Larus michahellis*
**223**	**128**	6	1	1	1	1	36	1	*Larus michahellis*
**224**	8	6	1	1	1	3	**124**	1	*Larus michahellis*
**225**	6	5	1	1	69	1	**123**	1	*Larus michahellis*
**226**	37	57	1	1	2	**147**	5	1	*Larus michahellis*
**227**	**128**	**121**	1	1	1	1	2	1	*Larus ridibundus*
**228**	37	4	**104**	1	1	3	6	1	*Larus ridibundus*
**229**	6	2	1	1	58	3	**127**	1	*Larus michahellis*
238	5	6	1	1	1	1	**2**	1	*Larus michahellis*
261	34	32	119	25	33	30	49	1	*Larus ridibundus*
**262**	103	2	1	1	**184**	3	44	1	*Larus michahellis*
**295**	8	6	1	1	58	1	2	2	*Larus ridibundus*
**296**	6	4	1	1	69	1	6	3	*Larus michahellis*
**297**	8	**152**	1	1	1	1	2	2	*Larus michahellis*
**298**	8	6	**120**	1	1	1	3	1	*Larus michahellis*
**299**	1	57	**121**	1	4	**172**	**159**	1	*Larus michahellis*
**306**	8	6	1	1	1	3	44	1	*Larus ridibundus*
**312**	8	6	1	1	1	3	2	3	*Larus michahellis*
**313**	84	6	1	1	1	1	36	1	*Larus michahellis*
**314**	8	6	1	1	58	119	36	1	*Larus michahellis*
**315**	8	1	1	1	1	1	**165**	2	*Larus michahellis*
**316**	103	62	1	1	1	3	44	1	*Larus michahellis*
**317**	7	5	1	1	69	1	2	1	*Larus michahellis*
**318**	8	2	1	1	1	3	**127**	1	*Larus michahellis*
**319**	6	5	1	53	2	5	6	1	*Larus michahellis*
**320**	8	6	1	1	4	1	36	2	*Larus michahellis*
**322**	6	5	1	1	**190**	1	6	1	*Larus michahellis*
**325**	8	6	1	53	58	1	2	1	*Larus michahellis*
**326**	103	6	1	1	4	1	2	1	*Larus michahellis*
**327**	7	1	1	53	1	127	2	1	*Larus michahellis*
	6	5	1	1	69	1	-	1	*Larus michahellis*
	-	6	1	1	1	1	36	1	*Larus michahellis*
	-	57	1	2	1	1	2	1	*Larus michahellis*
	**128**	-	1	1	1	-	36	1	*Larus michahellis*

**Table 2:** Distribution of MIC values among *Campylobacter lari* isolates (*n* = 43) from gulls’ cloacal swabs.

mg/L	CHL		ERY		GEN		CIP		TET		ETP	
512			0									
256			0									
128			0									
64	0		0						1			
32	0		0				0		0			
16	1		0		0		0		0			
8	0		0		0		5		0			
4	1		0		0		27		0		1	
2			0		2		11		0		0	
≤2	41											
1					27		0		1		0	
≤1			43									
0.5					12		0				1	
≤0.5									41			
0.25							0				0	
≤0.25					2							
0.12												
≤0.12							0				41	

Green—sensitive; red—resistant.

**Table 3:** MIC_50_ and MIC_90_ values and the percentage of *C. lari* isolates (*n* = 43) susceptible and resistant to different antimicrobials.

Antimicrobial Agent	MIC_50_ (mg/L)	MIC_90_ (mg/L)	(n/%) S	(n/%) R	EUCAST Epidemiological Cut-Off Value (ECCOF)
					R>
CHL	≤2	≤2	43/100	0/0	16
ERY	≤1	≤1	43/100	0/0	8
GEN	1	1	43/100	0/0	2 *
CIP	4	8	0/0	43/100	0.5
TET	≤0.5	≤0.5	42/97.7	1/2.3	2
ERTA	≤0.12	≤0.12	42/97.7	1/2.3	0.5 *

* [25].

## Data Availability

Data available on request.
